# Supplementary figures and images for: Structures of the mycobacterial membrane protein MmpL3 reveal its mechanism of lipid transport
Source: PLoS Biol. 2021 Aug 12;19(8):e3001370. doi: 10.1371/journal.pbio.3001370 (PMC8384468; doi:10.1371/journal.pbio.3001370)

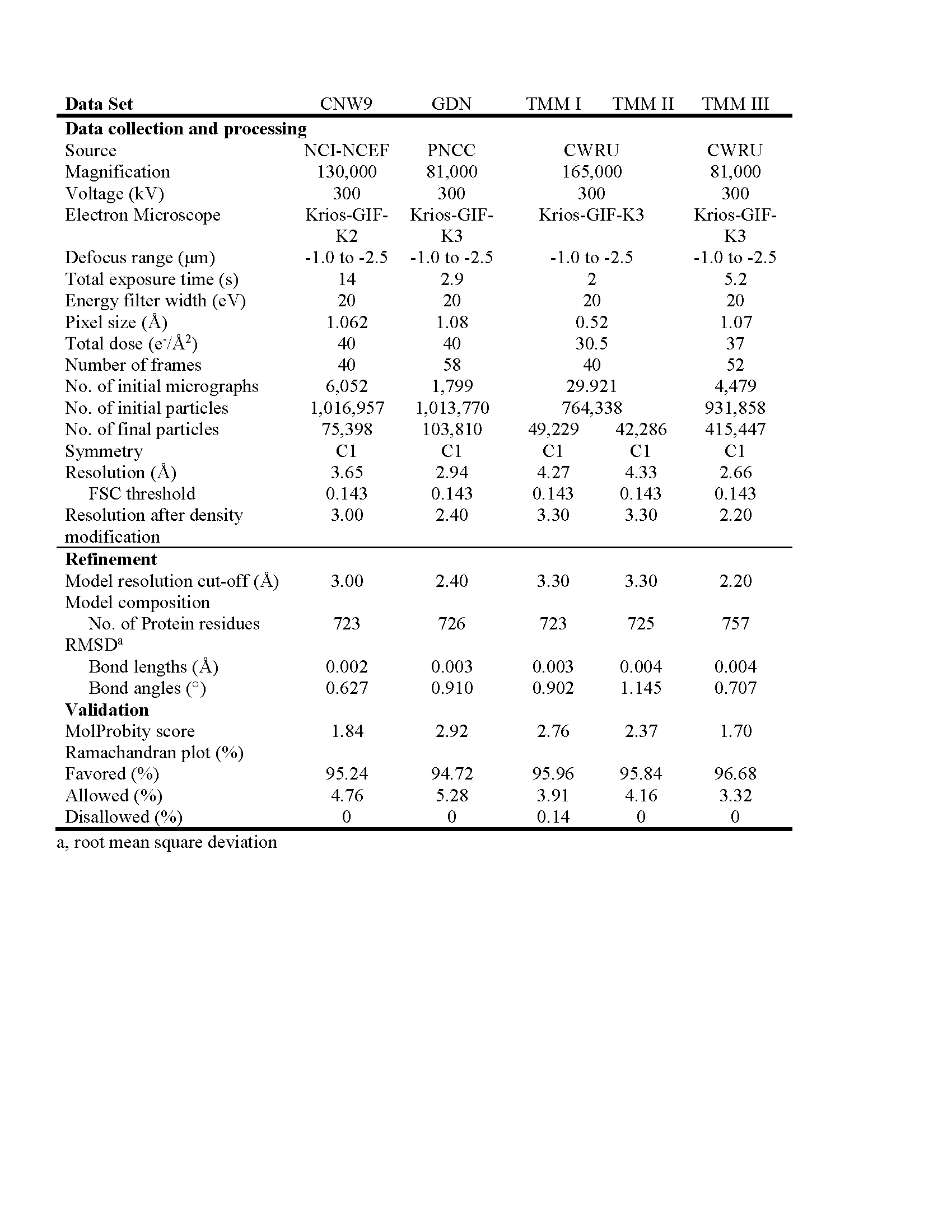

Supplement: S1 Table — (TIF) [file pbio.3001370.s001.tif]

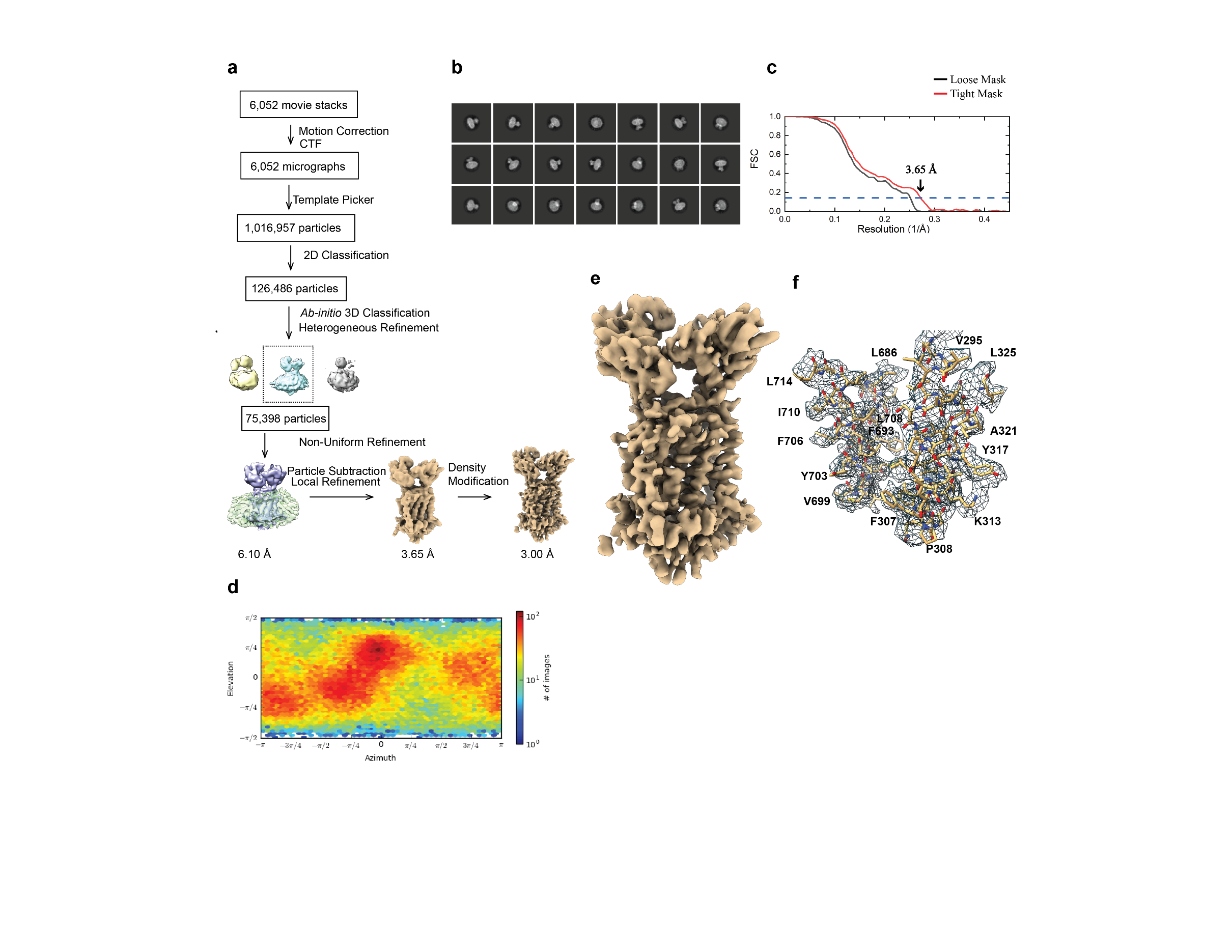

Supplement: S1 Fig — (a) Data processing flow chart with particle distributions. The black box indicates the particle class used for further refinement. (b) Representative 2D classes. (c) FSC curves. (d) Viewing direction distribution calculated in cryoSPARC for particle projections. This heat map shows number of particles for each viewing angle. (e) Sharpened cryo-EM map of the MmpL3 transporter viewed in the membrane plane. (f) Local EM density map of MmpL3. cryo-EM, cryo-electron microscopy; CTF, contrast transfer function; FSC, Fourier shell correlation; MmpL3, mycobacterial membrane protein large 3; MmpL3-ND, MmpL3-nanodisc. (TIF) [file pbio.3001370.s003.tif]

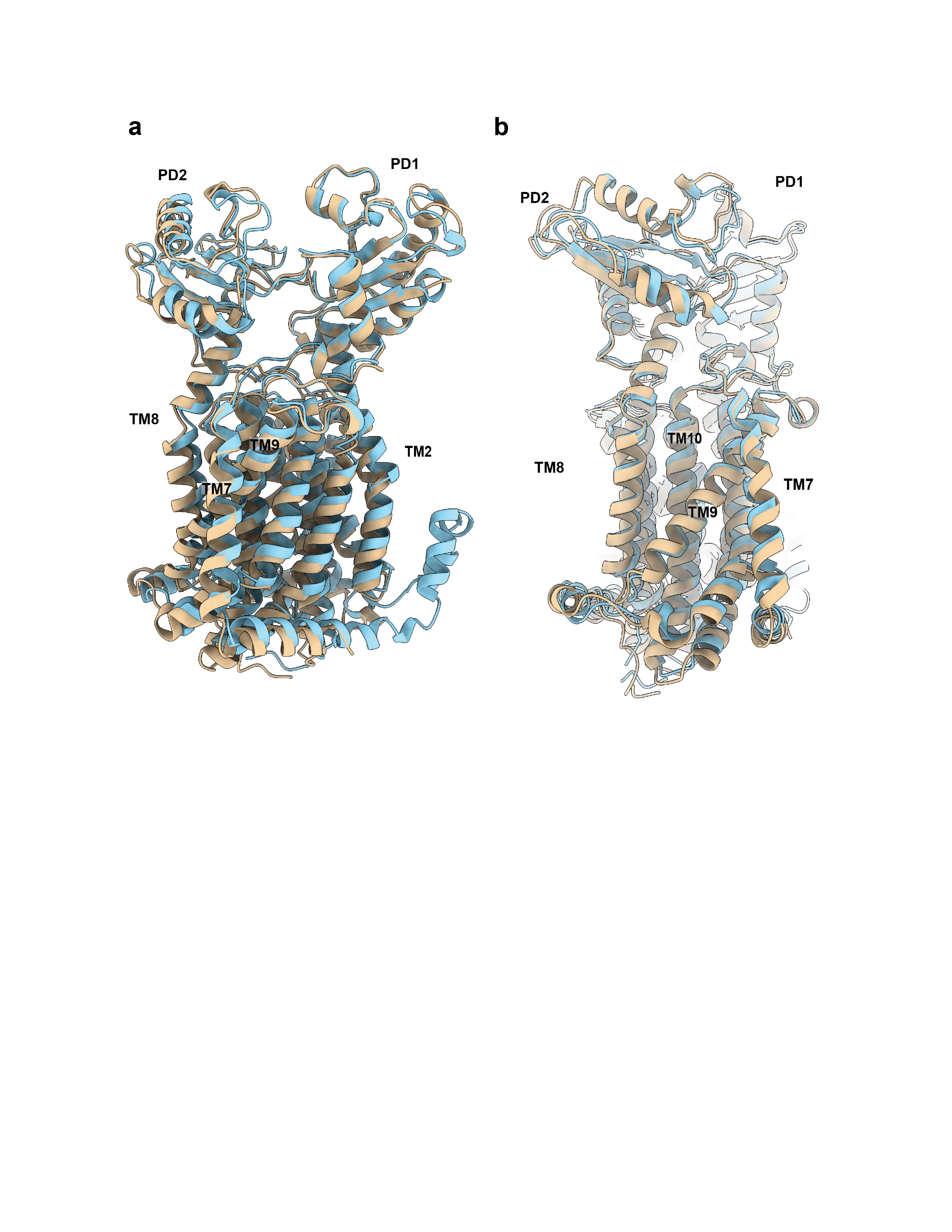

Supplement: S2 Fig — The superimposition gives rise to an RMSD of 1.5 Å (MmpL3-ND, orange; MmpL3773-PE, cyan). cryo-EM, cryo-electron microscopy; MmpL3-ND, MmpL3-nanodisc; RMSD, root mean square deviation. (TIF) [file pbio.3001370.s004.tif]

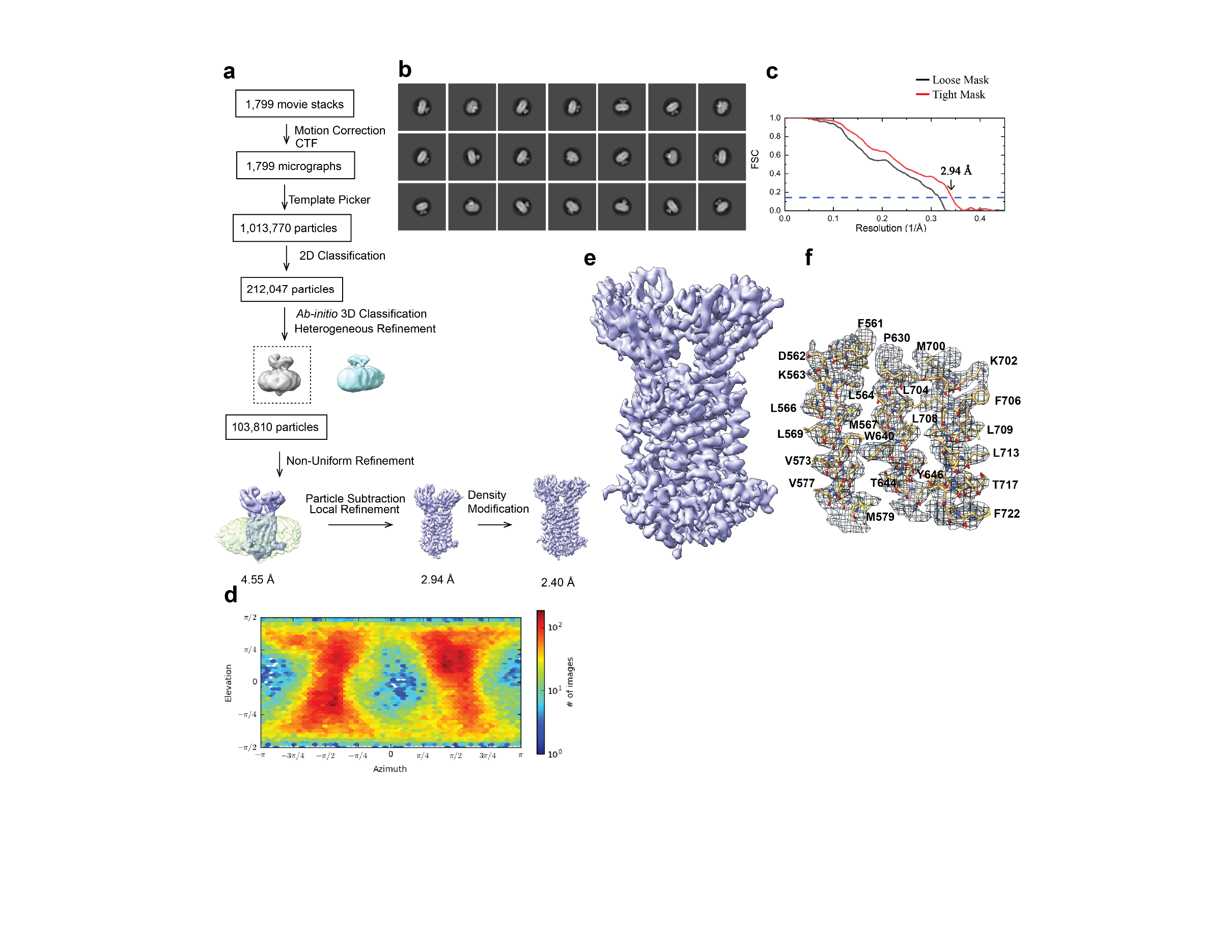

Supplement: S3 Fig — (a) Data processing flow chart with particle distributions. The black box indicates the particle class used for further refinement. (b) Representative 2D classes. (c) FSC curves. (d) Viewing direction distribution calculated in cryoSPARC for particle projections. This heat map shows number of particles for each viewing angle. (e) Sharpened cryo-EM map of MmpL3-GDN viewed in the membrane plane. (f) Local EM density map of MmpL3-GDN. cryo-EM, cryo-electron microscopy; CTF, contrast transfer function; FSC, Fourier shell correlation; MmpL3-GDN, MmpL3-glycol-diosgenin. (TIF) [file pbio.3001370.s005.tif]

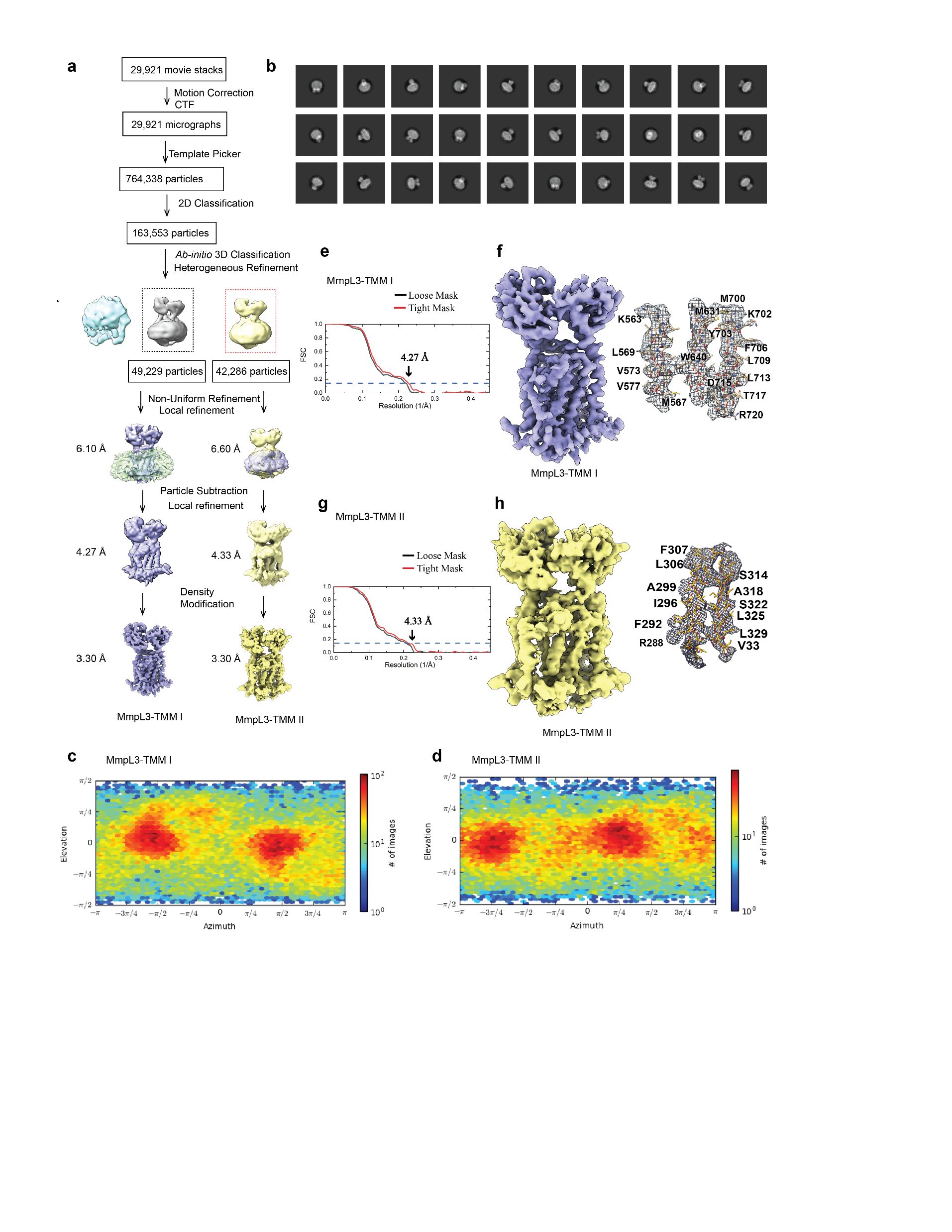

Supplement: S4 Fig — (a) Data processing flow chart with particle distributions. The black and red boxes indicate the particle classes used for further refinement of the structures of MmpL3-TMM I and MmpL3-TMM II, respectively. (b) Representative 2D classes. (c) Viewing direction distribution calculated in cryoSPARC for MmpL3-TMM I particle projections. The heat map shows number of MmpL3-TMM I particles for each viewing angle. (d) Viewing direction distribution calculated in cryoSPARC for MmpL3-TMM II particle projections. The heat map shows number of MmpL3-TMM II particles for each viewing angle. (e) FSC curves for the structure of MmpL3-TMM I. (f) Sharpened cryo-EM map of MmpL3-TMM I viewed in the membrane plane and its local EM density map. (g) FSC curves for the structure of MmpL3-TMM II. (h) Sharpened cryo-EM map of MmpL3-TMM II viewed in the membrane plane and its local EM density map. cryo-EM, cryo-electron microscopy; CTF, contrast transfer function; FSC, Fourier shell correlation; MmpL3-TMM, MmpL3-trehalose monomycolate. (TIF) [file pbio.3001370.s006.tif]

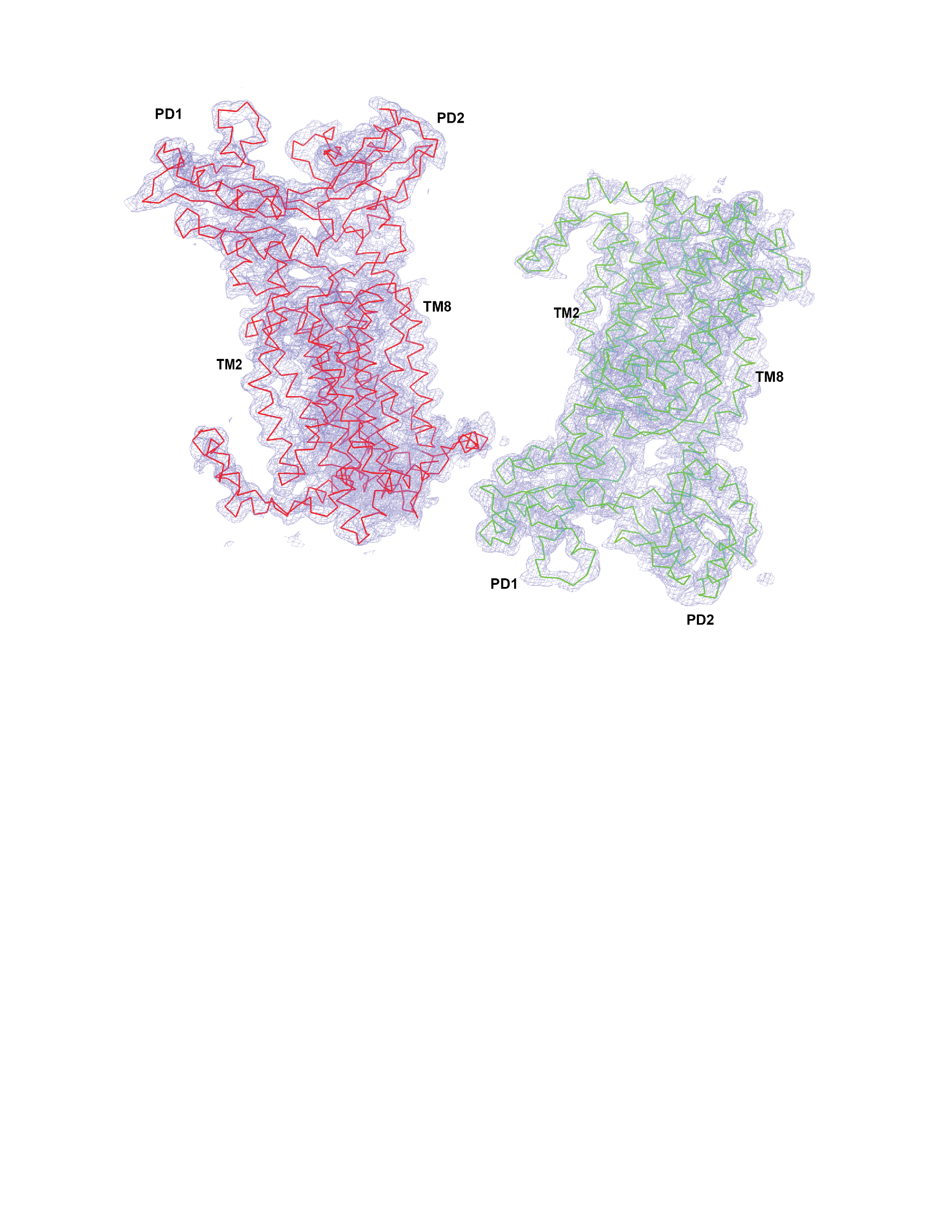

Supplement: S5 Fig — The electron density maps are contoured at 1.2 σ. The Cα traces of the 2 MmpL3 molecules in the asymmetric unit are in red (MmpL3773-T6D I) and green (MmpL3773-T6D II). These MmpL3 structures were determined by MR, utilizing the MmpL3773-PE structure (pdb id: 6OR2) as a search model. MmpL3, mycobacterial membrane protein large 3; MR, molecular replacement. (TIF) [file pbio.3001370.s007.tif]

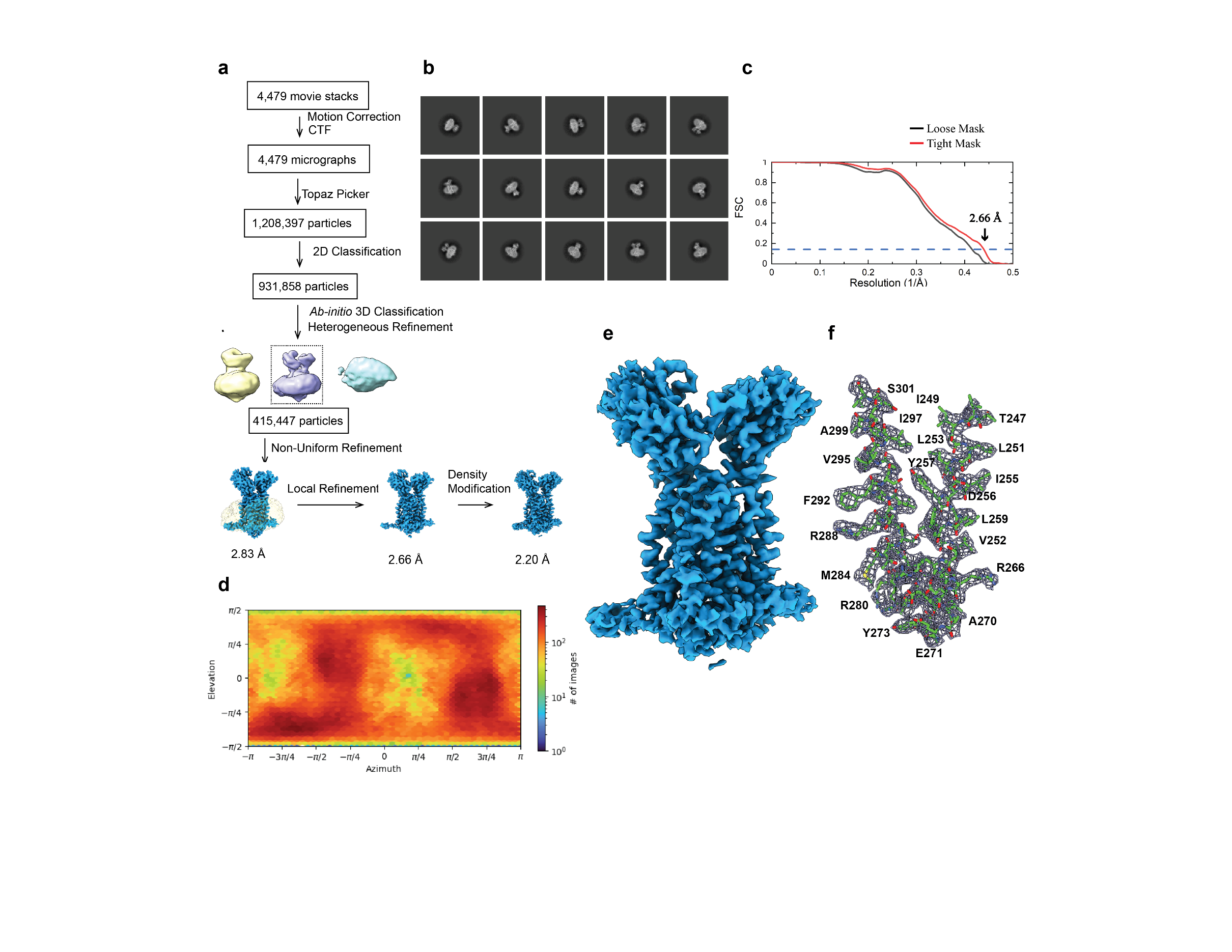

Supplement: S6 Fig — (a) Data processing flow chart with particle distributions. The black box indicates the particle class used for further refinement. (b) Representative 2D classes. (c) FSC curves. (d) Viewing direction distribution calculated in cryoSPARC for particle projections. This heat map shows number of particles for each viewing angle. (e) Sharpened cryo-EM map of MmpL3-TMM III viewed in the membrane plane. (f) Local EM density map of MmpL3-TMM III. cryo-EM, cryo-electron microscopy; CTF, contrast transfer function; FSC, Fourier shell correlation; MmpL3-TMM, MmpL3-trehalose monomycolate. (TIF) [file pbio.3001370.s008.tif]

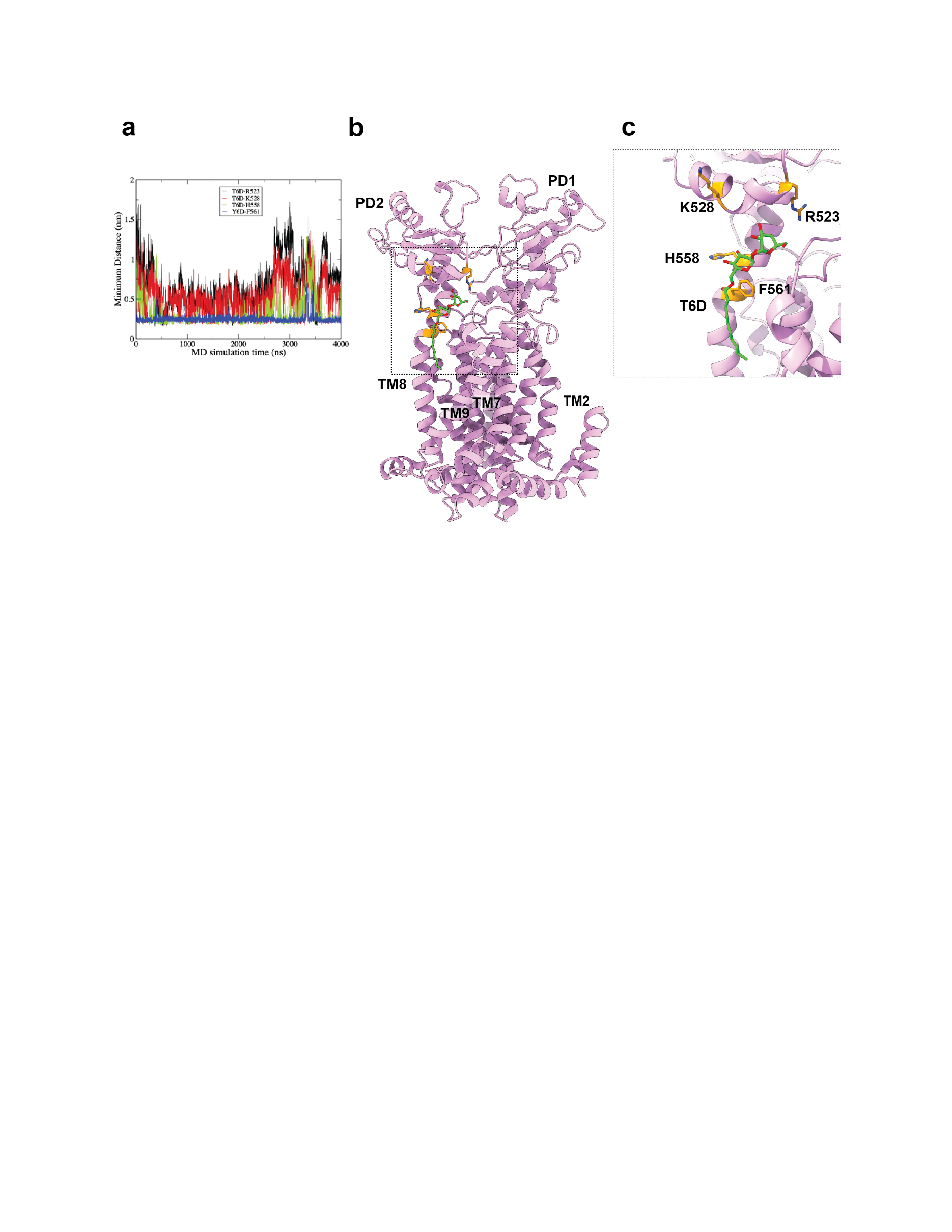

Supplement: S7 Fig — (a) Minimum distances between T6D and these 4 residues (T6D-R523, black; T6D-K528, red; T6D-H558, green; and T6D-F561, blue). (b and c) The locations of residues R523, K528, H558, and F561. Residues R523, K528, H558, and F561 are shown as yellow sticks. The bound T6D molecule is in green sticks. The secondary structural elements of MmpL3 are colored pink. MD, molecular dynamics; MmpL3, mycobacterial membrane protein large 3; T6D, trehalose 6-decanoate. (TIF) [file pbio.3001370.s009.tif]

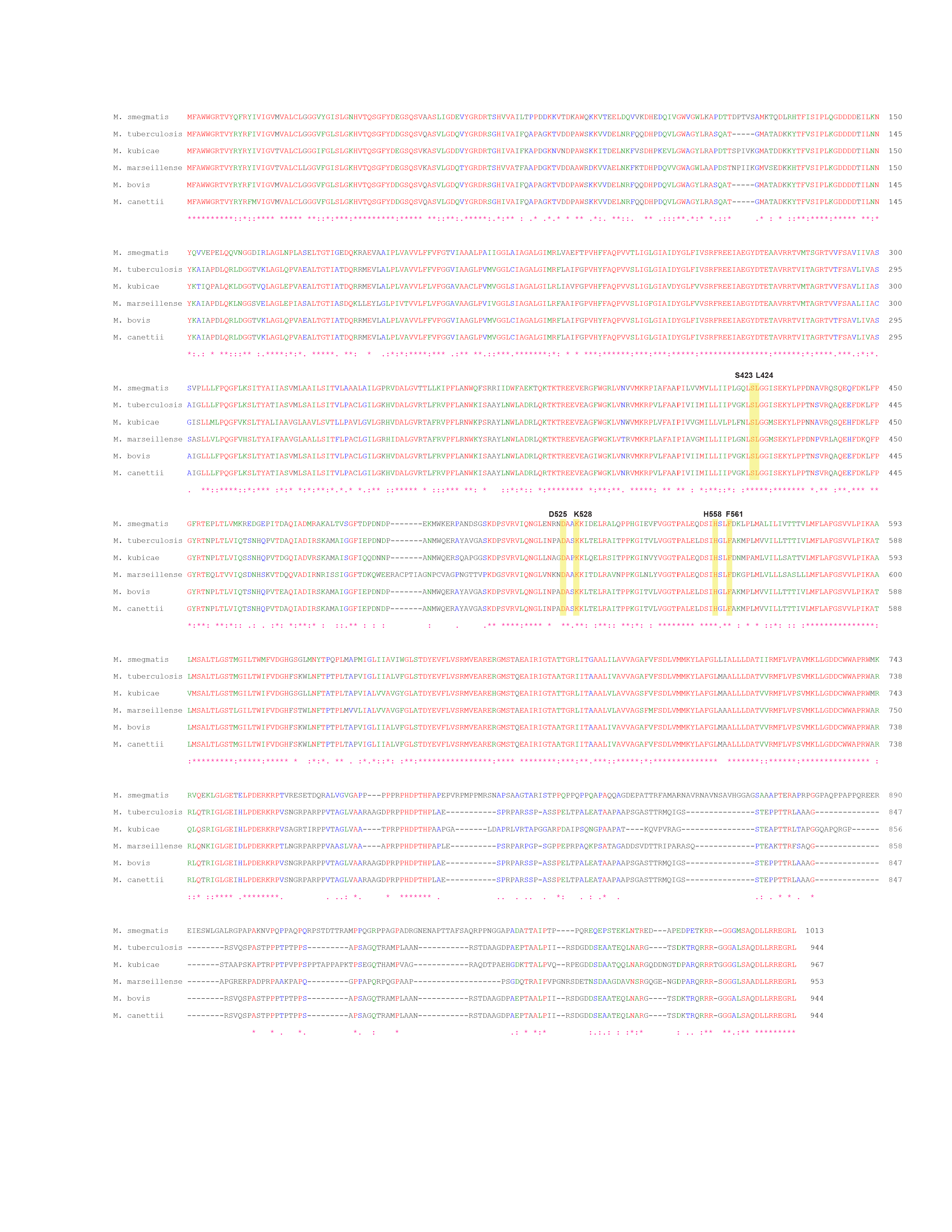

Supplement: S8 Fig — This alignment suggests that the gate residues S423, L424, and D525, as well as the residues K528, H558, and F561 that interact with T6D are conserved among these transporters. MmpL3, mycobacterial membrane protein large 3; T6D, trehalose 6-decanoate. (TIF) [file pbio.3001370.s010.tif]

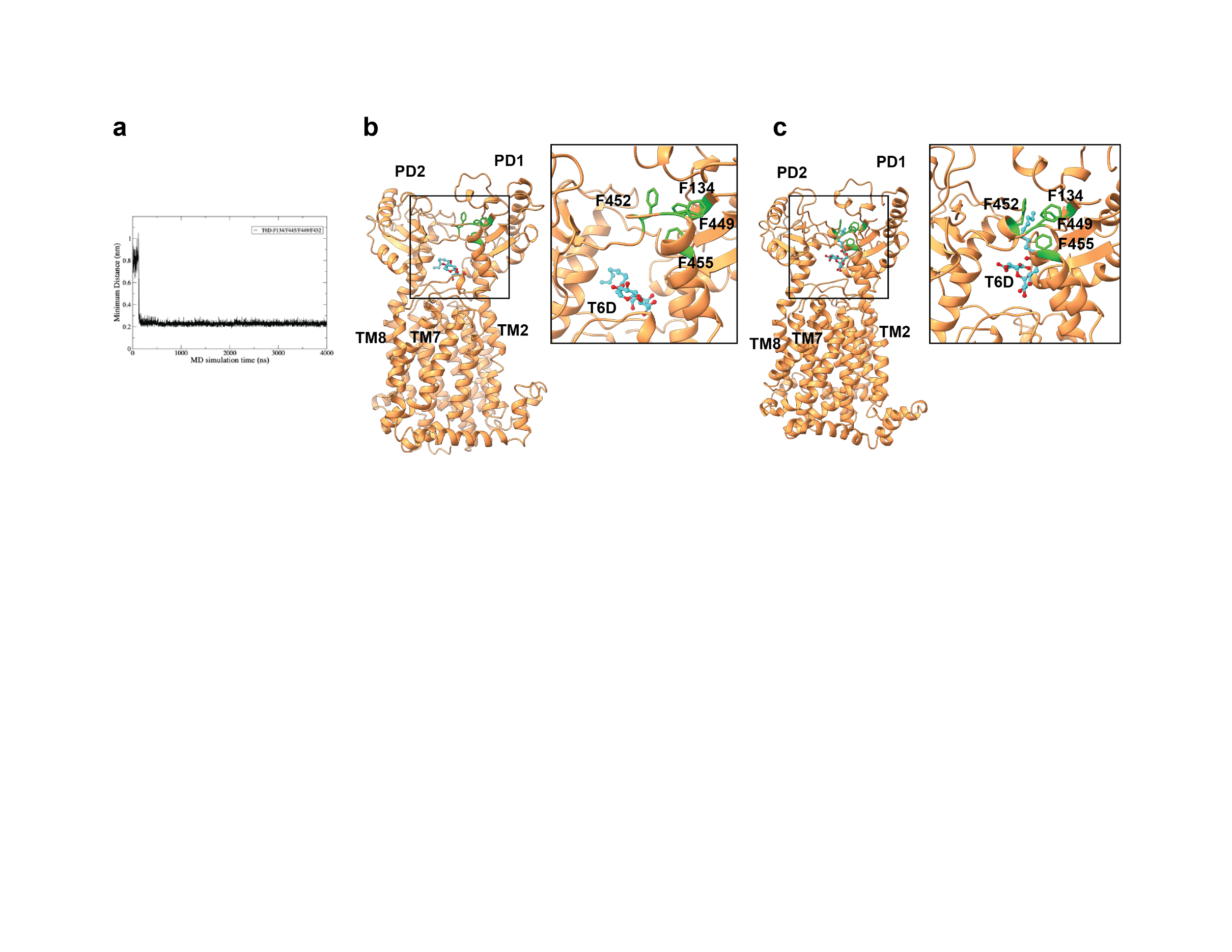

Supplement: S9 Fig — (a) MD simulations indicate the minimum distance between T6D and the Phe cluster as a function of time. (b) The locations of T6D (especially the hydrocarbon tail) and the Phe cluster (F134, F449, F452, and F455) at 0 ns. The T6D molecule is colored cyan. Residues F134, F449, F452, and F455 are colored green. The secondary structural elements of MmpL3 are colored orange. (c) The locations of T6D (especially the hydrocarbon tail) and the Phe cluster (F134, F449, F452, and F455) at 4,000 ns. The T6D molecule is colored cyan. Residues F134, F449, F452, and F455 are colored green. The secondary structural elements of MmpL3 are colored orange. MmpL3, mycobacterial membrane protein large 3; T6D, trehalose 6-decanoate. (TIF) [file pbio.3001370.s011.tif]

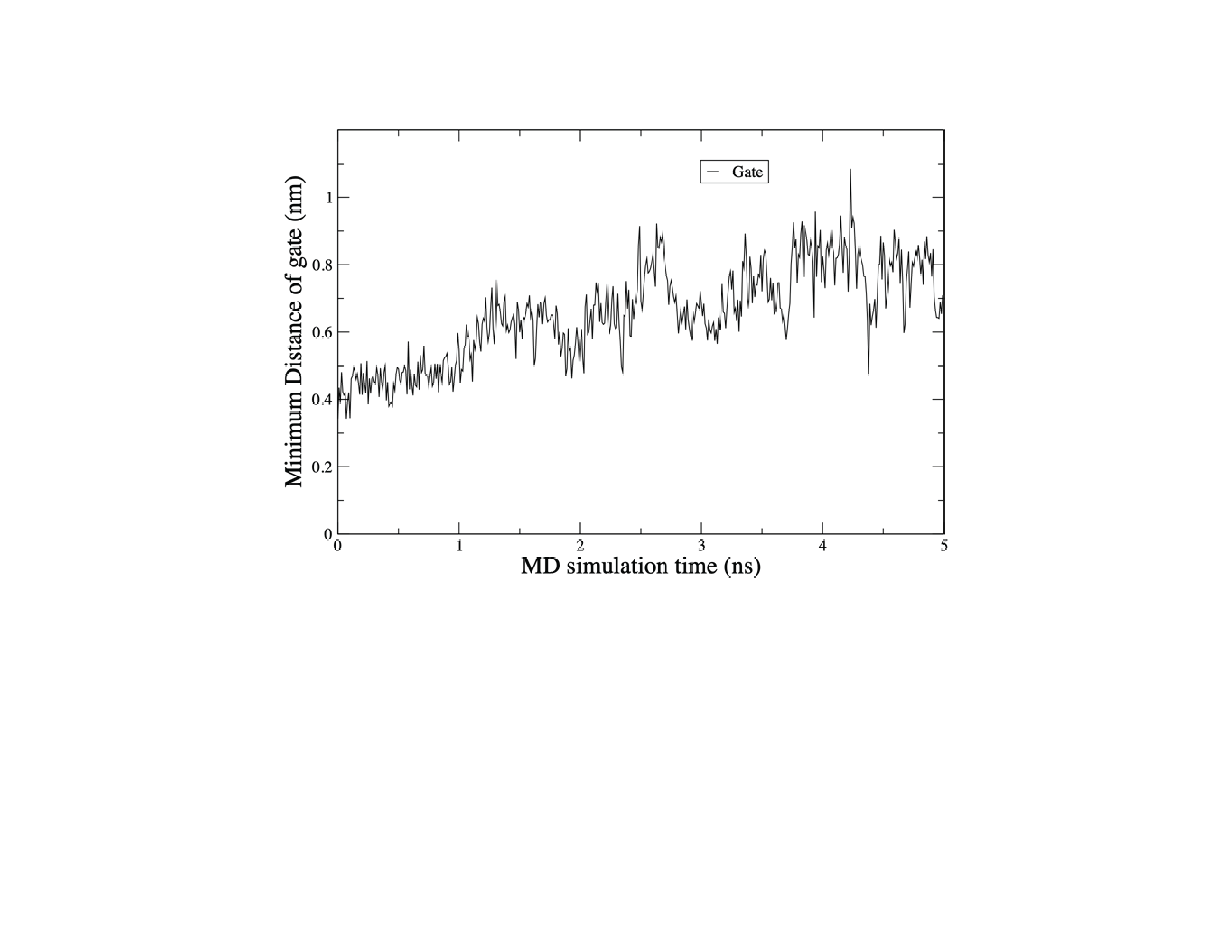

Supplement: S10 Fig — This figure indicates the minimum distance of the gate as function of time during TMD simulations. MD, molecular dynamics; TMD, target MD. (TIF) [file pbio.3001370.s012.tif]
