# Supplementary material for: Structures of the mycobacterial membrane protein MmpL3 reveal its mechanism of lipid transport
Source: PLoS Biol. 2021 Aug 12;19(8):e3001370. doi: 10.1371/journal.pbio.3001370 (PMC8384468; doi:10.1371/journal.pbio.3001370)
Supplement: Attachment — Submitted filename: Point-by-point response to reviewer MmpL3.docx [file pbio.3001370.s013.docx]

We are submitting a revised version of our manuscript entitled “Structures of MmpL3 illuminate a mechanism of lipid transport.” Two of the referees have raised several important points that we have now addressed in this revision of the paper.

Our revised paper has an expanded scope. An important set of experiments using twice the original concentration of TMM in nanodiscs has allowed us to solve the cryo-EM structure of the MmpL3-TMM complex to a resolution of 2.20 Å. This important structure has also permitted us to elucidate how MmpL3 specifically interacts with TMM and how it shuttles this lipid molecule to the cell wall.

We also performed computational alanine scanning experiments on residues K528, H558 and F561 of the MmpL3 transporter to understand the importance of these residues on T6D binding. We found that these alanine mutants bind the substrate T6D weaker in comparison with wild-type MmpL3.

Our point-by-point response to the referees’ comments follows this letter.

The changes are highlighted with blue color in the markup revised manuscript.

We believe that this work is an outstanding scientific contribution, reporting molecular structures of the most important transporter, MmpL3, within the MmpL family. To define the molecular mechanism of intracellular TMM translocation in *Mycobacterium* species, we report seven structures of the *M. smegmatis* MmpL3 transporter, both in the apo form and bound with TMM or other lipid moieties, by single-particle cryo-electron microscopy (cryo-EM) and X-ray crystallography. Combined with calculated results from molecular dynamics (MD) and target MD simulations, we reveal a lipid transport mechanism that involves a coupled movement of the periplasmic domain and transmembrane helices of the MmpL3 transporter that facilitates the shuttling lipids to the mycobacterial cell wall. We believe that this work is highly significant and will make an impact on the mycobacterial field.

Sincerely,

REVIEWS:


Reviewer #1: Structural Biology of transports

Reviewer #2: Structural biology of membrane proteins


Reviewer #1: The mycobacterial membrane transport protein MmpL3 is of both fundamental and practical significance, as it is essential for transport of trehalose monomycolate (TMM), a precursor for several components of the mycobacterial outer membrane. It is a target of several tuberculosis (TB) drugs, and a validated target for future drug discovery/development. Although no M. tuberculosis (Mtb) MmpL3 structures have been determined to date, Mtb MmpL3 can be complemented by the MmpL3 ortholog from M. smegmatis (Msm). The first structures of Msm MmpL3 were determined in 2019, first by Zhang et al. (Cell 176:636), and then shortly thereafter, also in 2019, by the Yu group (PNAS 116:11241), who are the authors of this PLoS Biology submission. This manuscript presents and describes six structures, determined by x-ray crystallography or cryoelectron microscopy (these latter being the first cryoEM structures of MmpL3), accompanied by detailed analysis of the structures along with
several types of atomistic molecular dynamics (MD) simulations. The structures presented differ in a variety of ways, and the goal of the subsequent analysis, and especially of the MD simulations, is to infer/suggest/hypothesize transport mechanisms of MmpL3 based upon these structural "inputs."

The PI has a distinguished record of excellence in membrane protein structural biology, with a focus upon x-ray crystallography of bacterial AcrB transporters. The MmpL3 structures presented are a welcome addition and will likely be of significant future utility in the design of functional experiments (including MmpL3 mutagenesis) to obtain detailed mechanistic insights into the transport cycle of this important mycobacterial transporter. However, to this Reviewer, the mechanism suggested here, of coupled movement of the MmpL3 periplasmic domain with its transmembrane (TM) helices, is rather highly speculative and not compellingly demonstrated by the combination of structures, structural analysis and MD simulations presented. Some specific questions/concerns follow.

We very much appreciate this reviewer’s responses, especially indicating that the MmpL3 structures presented are a welcome addition and will likely be of significant future utility in the design of functional experiments. We also would like to thank this reviewer for the suggestion to change the concentration of TMM in nanodiscs. In this revised manuscript, we have added an important cryo-EM structure of the MmpL3-TMM complex at 2.20 Å resolution. We obtained this important structure by following this reviewer’s suggestion to increase the concentration of TMM by twofold.

1. Previous MmpL3 structures are insufficiently discussed, and bear upon the analysis presented. The structures of Zhang et al. (Cell 176:636) include MmpL3 with bound Tb drugs/drug candidates, where these appear to be located in the lumen of the transporters. Do the authors of this manuscript consider this site to also be in the transport/permeation pathway of the TMM? Even more critically, the authors' previous MmpL3 structural publication (PNAS 116:11241) indicates that the presence of bound detergent (DDM) and bound lipid (PE). To this Reviewer's eye, the DDM site looks like the "T6D1" site & the PE site looks like the "T6D2" site in the structures presented in this manuscript, respectively. Therefore, what is (most) new/significant about the sites presented here?

In view of the seven structures of MmpL3 in this manuscript, we have not observed any cavities or pockets formed within the transmembrane region. Therefore, it is unlikely that MmpL3 would utilize the inhibitor-binding site documented in the Cell paper to translocate TMM lipids. A new paragraph “The X-ray structures of MmpL3 bound with a variety of inhibitors indicate that TMs 4, 5, 6, 10, 11 and 12 of this transporter participate in forming a large binding pocket to accommodate for the binding of these inhibitors. Each of these small molecules were found within the middle of the transmembrane domain, presumably disrupting the proton-relay network of the transporter. However, our cryo-EM and X-ray structures of MmpL3, either alone or bound with lipid moieties, depict that the TM helices are closely packed and do not appear to form a cavity or pocket to bind substrates. There is a chance that the inhibitor-binding site at the transmembrane domain could open up only in the presence of these inhibitors. Therefore, it is unlikely that the MmpL3 transporter would utilizes this site as a pathway to shuttle lipid moieties” has been added in the “Discussion” section (p.16) of the revised manuscript to address this.

2. Are the structural differences observed among cryoEM nanodisc MmpL3 structures due to membrane variation caused by addition of TMM to the "membrane mimetic", rather than arising from functional conformational substates of the transporter? MmpL3 nanodiscs (NDs) were prepared in the absence and presence of Msm glycolipids (btw, details on preparation/sourcing of these glycolipids appears to be absent). No (ordered) TMM is seen in the cryoEM structures of the +TMM ND samples, which seems suggestive of TMM mixing with the E. coli lipids to form the bilayer region encircled by the molecular scaffold protein (MSP), rather than binding to MmpL3 (T6D was seen in the detergent-solubilized x-ray structure). Were there any characteristics of the NDs, such as diameter or thickness, which were different +/- TMM? Future experiments utilizing a wider range of TMM, in multiple concentration steps, with effect upon observed structures could help sort this out. Also, experiments with T6D
instead of TMM could be very informative in ND cryoEM structures, in terms of actually showing up as bound to MmpL3. The variation of MmpL3 in ND vs. GDN is suggestive of different membrane mimetics affecting structures.

We thank this reviewer’s comments. We followed this reviewer’s suggestion and increased the concentration of TMM in nanodiscs by twofold to repeat the cryo-EM experiment. Fortunately, we obtained the cryo-EM structure of the MmpL3-TMM complex using this approach. The structure of the MmpL3-TMM was solved to a resolution of 2.20 Å. Two TMM molecules were identified to bind within the MmpL3 transporter. A new section of the “Cryo-EM structure of the MmpL3-TMM complex” (p.10-12) has been included in this revised manuscript to describe the structure of this MmpL3-TMM complex.

3. A primary result obtained from the structures is that periplasmic subdomain PD2 is in variable positions relative to periplasmic subdomain PD1. This is a solid structural/empirical observation. However, what follows from this, to this Reviewer, is very highly speculative. While the speculative nature of this is mentioned reasonably well at some places in the manuscript, some of the assumptions (and conclusions) are presented with a much higher level of certainty.
- "This rigid-body rotation may allow for the expansion of the central periplasmic cavity ..."
- "These two major conformational changes may couple with each other ..."
- "... these six MmpL3 structures can be interpreted as snapshots of various transient states ..."
(this last statement seems especially unsubstantiated.)
- "It is likely that S423 and N254 form a gate of the channel that is able to open and close ..."
The relation of the MD results, at shorter length scales, to the larger-scale structural variations observed and the couplings mentioned, are not connected in a way that is clear to this Reviewer. They seem more like rather independent results. This discussion could be shortened and clarified. It would be useful to have specific functional predictions made for the effects of specific mutations that are suggested by the structures, analysis and simulations. Also, multiple sequence alignment (MSA) of mycobacterial MmpL3 orthologs could add to this.

We believe that the speculations in our discussion are reasonable, as our structural data (both cryo-EM and X-ray crystallography) are in strong agreement with those of MD simulations. Nonetheless, the two paragraphs at the beginning of the “Computational simulations” section have been significantly shortened and combined into one single paragraph in the revised version of the manuscript, following this reviewer’s suggestion. The statement “This rigid-body rotation may allow for the expansion of the central periplasmic cavity to accommodate for lipid binding” has been removed. The statement “These two major conformational changes may couple with each other to facilitate the translocation of lipids across the transporter” has been modified to “It is possible that these two major conformational changes could couple with each other to facilitate the translocation of lipids across the transporter”. In addition, the statement “It is likely that S423 and D525 form a gate of the channel that is able to open and close in order to advance the transport cycle” has been rephrased to “There is a chance that S423 and D525 may form a gate of the channel that is able to open and close in order to advance the transport cycle” in this revised manuscript.

We also included a new figure for alignment of protein sequences of different mycobacterial MmpL3 transporters in this revised manuscript (Fig. S8). Based on the sequence alignment, it appears that K528, H558 and F561, are conserved among the mycobacterial species. We then used the approach of molecular mechanics generalized Born surface area (MM-GBSA) to conduct computational alanine scanning experiment on residues K528, H558 and F561 of the MmpL3 transporter to study the effects of mutation on the binding affinity of the T6D ligand. We observed that the binding affinity for T6D is the strongest in the wild-type MmpL3 transporter. In comparison with wild-type MmpL3, the relative binding affinities for T6D in the K528A, H558A and F561A mutants are 0.264, 2.682, and 3.801 kcal/mol weaker. These data have been included (on p.14) of this revised version of the manuscript.

4. How are cavity volumes calculated? How are structures superposed, for rmsd?

The cavity volumes were calculated using the program CASTp 3.0. Statements indicating that these volumes were computed using the program CASTp 3.0 have been included in this revised manuscript.

We superimposed 710 Cα atoms of two different MmpL3 structures to obtain these r.m.s.d. values. This has also been included in this revised manuscript.

Reviewer #2: The MmpL3 is an essential protein in mycobacterium. It is required for shuttling TMM from the cytoplasmic membrane out to the outer membrane of mycobacterium. The authors Su et al here present 6 different structures of MmpL3; in detergent, in nanodisc, with and without ligands. Based on these structures the authors are able to speculate as to how substrate is bound and transferred from the lipid bilayer to the periplasmic binding pocket of the protein. The work is complemented with MD simulations further illustrating the movements within the protein upon substrate binding. Overall, I think this is a comprehensive and very informative study, that I think would be a great addition to PLOS Biology.

We thank this reviewer for the positive support, indicating that this work would be a great addition to PLOS Biology.

I do have a couple of suggestions that I think would make the manuscript easier to follow, and a couple of questions for the authors to address.
*       A schematic of the process of TMM translocation would be very helpful for the reader. For example, a cartoon drawing showing the inner and outer membrane, the position of MmpL3 and path of TMM.

This is a great suggestion. A new schematic (Fig. 8) indicating the process of TMM translocation has been added in this revised manuscript.

*       In the text when the authors describe the protein expression and purification they say "The MmpL3 membrane protein was overproduced in E. coli BL21(DE3)ΔacrB cells and purified using a Ni2+-affinity column. We delipidated and reconstituted the purified MmpL3 transporter into lipidic nanodiscs." After looking through their methods I believe they delipidate and solubilize before Ni affinity purification and the sentence should therefore be "The MmpL3 membrane protein was expressed in E. coli BL21(DE3)ΔacrB cells, solubilized using detergent and purified using a Ni2+-affinity column. The purified protein was reconstituted into lipidic nanodiscs."

Yes, this reviewer is correct. The statements “The MmpL3 membrane protein was overproduced in *E. coli* BL21(DE3)*ΔacrB* cells and purified using a Ni^2+^-affinity column. We delipidated and reconstituted the purified MmpL3 transporter into lipidic nanodiscs.” at the beginning of the “Results” section (p.5) have been modified to “The MmpL3 membrane protein was overproduced in *E. coli* BL21(DE3)*ΔacrB* cells. We delipidated and purified this membrane protein using a Ni^2+^-affinity column. We then reconstituted the delipidated, purified MmpL3 transporter into lipidic nanodiscs.” in this revised version of the manuscript.

*       When the authors mention RMSD they should include if this is an all atom RMSD or a backbone.

We superimposed 710 Cα atoms of two different MmpL3 structures to calculate these r.m.s.d. values. This has been mentioned in several places of this revised manuscript to indicate how we obtained these values.

*       The conformational change between MmpL3 in ND and detergent is relatively small, but I was surprised to see the conformational changes that were observed all seem to be away from the membrane, why do they authors think this conformational change is observed?

Yes, we observed that the most obvious difference among different MmpL3 structures is the relative location of PD2 in relation to PD1. This observation is in good agreement with results from MD simulations that the first eigenvector, which depicts the most important motion extracted from the MD simulation trajectory, corresponds to a rotational motion of the periplasmic subdomain PD2 in relation to PD1. However, we also found from both the structural and computational data that the transmembrane helices TM7-TM12 also shift their locations, seemingly to accompanying the rotational motion of PD2 (Fig. 6 of the revised manuscript). It is possible that these two major conformational changes could couple with each other to facilitate the translocation of lipids across the transporter. In addition, the periplasmic domain and transmembrane domain of MmpL3 are connected together via several flexible loops. The flexible loops should facilitate the flexibility of periplasmic domain in relation to the transmembrane domain.

*       In the discussion the authors propose a model where TMM comes into the MmpL3 protein from the periplasmic side of the cytoplasmic membrane and is transported into the periplasmic pocket. This model would suggest that MmpL3 is in fact not a flippase. I would like a discussion of this in the paper, is there another flippase associated?

We actually think that MmpL3 is a flippase. However, our MmpL3 structures may capture different conformational states of the transporter after the TMM lipid has been flipped from the inner leaflet to outer leaflet of the membrane. We have added a new paragraph in the “Discussion” section (p.17) of this revised manuscript to clarify this. We have also included the flipping process of the TMM lipid as the first step in our new schematic diagram (Fig. 8).

*       It seems to me you have some stretching of the density in the ND structure. It would be nice to include an Euler angle distribution of all particles used in the final map reconstruction in the supplementary figures.

The Euler angle distributions of all particles have been included in the supplementary materials (Fig. S1d, Fig. S3d, Fig. S4c-d and Fig. S6) of this revised version of the manuscript.

Minor comment:
Typo in supplementary figure 1, 3 and 4, I should be refinement not refinment.

Thank you for this reviewer. The typos in Figs. S1, S3 and S4 have been fixed. The word refinement has been corrected.

It would be helpful with more references to figures and more precise references such as instead of Fig 3 say Fig 3a and so on.

Yes, we have redone these figure references. For example, we referred to Fig. 1a, Fig. 1b, etc. instead of Fig. 1.
