# Supplementary material for: Structures of the mycobacterial membrane protein MmpL3 reveal its mechanism of lipid transport
Source: PLoS Biol. 2021 Aug 12;19(8):e3001370. doi: 10.1371/journal.pbio.3001370 (PMC8384468; doi:10.1371/journal.pbio.3001370)
Supplement: Attachment — Submitted filename: Point-by-point response revised revised.docx [file pbio.3001370.s014.docx]

We are submitting a revised version of our manuscript. The title of the manuscript has been modified to “Structures of the mycobacterial membrane protein MmpL3 reveal its mechanism of lipid transport”, following the editor’s suggestion.

All PDB and EMDB IDs are included in the manuscript.

We believe that this work is an outstanding scientific contribution, reporting molecular structures of the most important transporter, MmpL3, within the MmpL family. To define the molecular mechanism of intracellular TMM translocation in *Mycobacterium* species, we report seven structures of the *M. smegmatis* MmpL3 transporter, both in the apo form and bound with TMM or other lipid moieties, by single-particle cryo-electron microscopy (cryo-EM) and X-ray crystallography. Combined with calculated results from molecular dynamics (MD) and target MD simulations, we reveal a lipid transport mechanism that involves a coupled movement of the periplasmic domain and transmembrane helices of the MmpL3 transporter that facilitates the shuttling lipids to the mycobacterial cell wall. We believe that this work is highly significant and will make an impact on the mycobacterial field.

Sincerely,
